# Case report: Quadruple primary malignant neoplasms including esophageal, ureteral, and lung in an elderly male

**DOI:** 10.1515/biol-2022-0465

**Published:** 2022-09-16

**Authors:** Long Wan, Feng-yan Yin, Hai-hua Tan, Li Meng, Jian-hua Hu, Bao-rong Xiao, Zhao-feng Zhu, Ning Liu, Huan-peng Qi

**Affiliations:** Department of Oncological Radiotherapy, Tai’an Central Hospital, No. 29 Longtan Road, Tai’an 271000, Shandong Province, China; Department of Thyroid Surgery, Tai’an Central Hospital, Tai’an 271000, Shandong Province, China

**Keywords:** multiple primary malignant neoplasm, overall survival, esophageal squamous cell carcinoma, urinary tract urothelial carcinoma, small cell lung cancer, lung squamous cell carcinoma

## Abstract

Multiple primary malignant neoplasms (MPMNs) are defined as multiple tumors with different pathogenic origins. MPMNs are rare, but the morbidity rate is on the rise. With the development of anti-tumor treatments, such as targeted therapy and immunotherapy, the overall survival of cancer patients has been significantly prolonged, leading to an increased number of patients with MPMNs. A crucial aspect of MPMNs management is deciding how to schedule further treatments according to individual tumor risk. This process involves a multidisciplinary physician team to ensure favorable outcomes. Herein we report a 60-year-old male who developed four different malignancies, including esophageal squamous cell carcinoma, upper urinary tract urothelial carcinoma, mediastinal small cell lung cancer, and left lung squamous cell carcinoma over 20 years and received appropriate treatment of each cancer with long survival.

## 1 Introduction

Multiple primary malignant neoplasms (MPMNs), also known as multiple primary cancers, are defined as multiple tumors with different pathogenic origins [1,2]. According to the interval of time between the first and second primary tumors, MPMNs are divided into metachronous (≥6 months) and simultaneous MPMNs (<6 months) [3]. The number of cancer patients with long-term survival is increasing, and the risk of having second or more tumors is on the rise because of advances in diagnosis and treatment [4]. Although there are a number of reports on the occurrence of two primary malignancies, there are few reports on three or more primary malignancies. We searched Pubmed and found that there have been 58 reports of quadruple carcinomas since the first report of a quadruple carcinoma in 1962 [5]. There are no standard treatment guidelines or uniform expert consensus for MPMNs. Therefore, the treatment is varied and the prognosis is poor. Herein we reported a patient with four primary metachronous cancers including esophageal squamous cell carcinoma (ESCC), upper urinary tract urothelial carcinoma (UTUC, bladder metastasis 1 year later), mediastinal small cell lung cancer (SCLC), and left lung squamous cell carcinoma (LUSC). After sequential diagnosis and appropriate treatment in multiple departments, the patient had a long-term survival.

## 2 Case presentation

**Figure 1:**
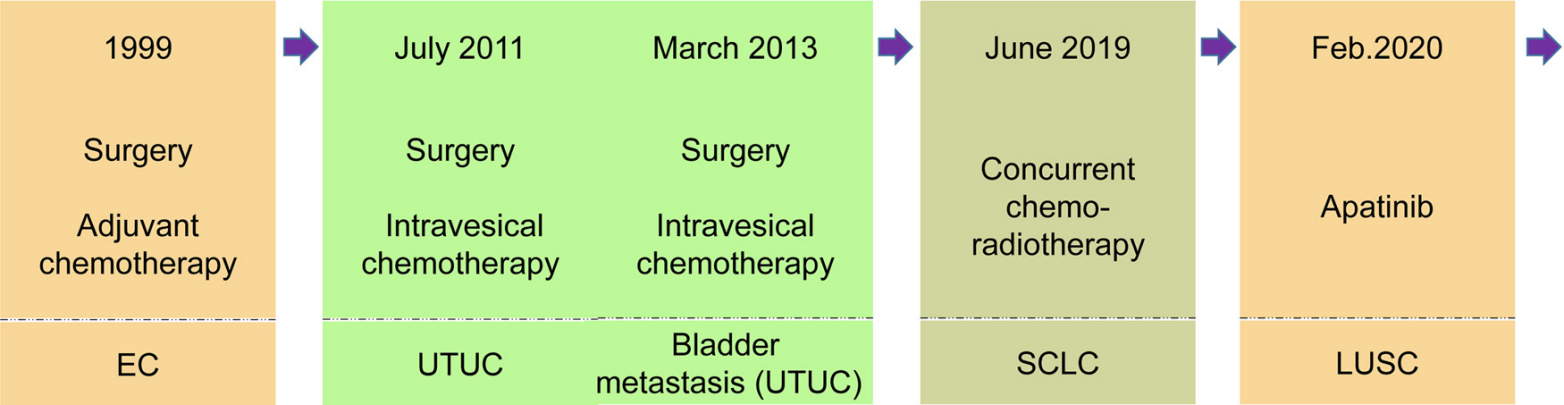
Flow chart of medical history.

Between 1998 and 2020, a case report described a patient with four primary tumors including ESCC, UTUC, SCLC, and LUSC with separate pathologic features and explored sustainable and reasonable management of multiple primary tumors, who had a long overall survival (Figure 1).

**Figure 2:**
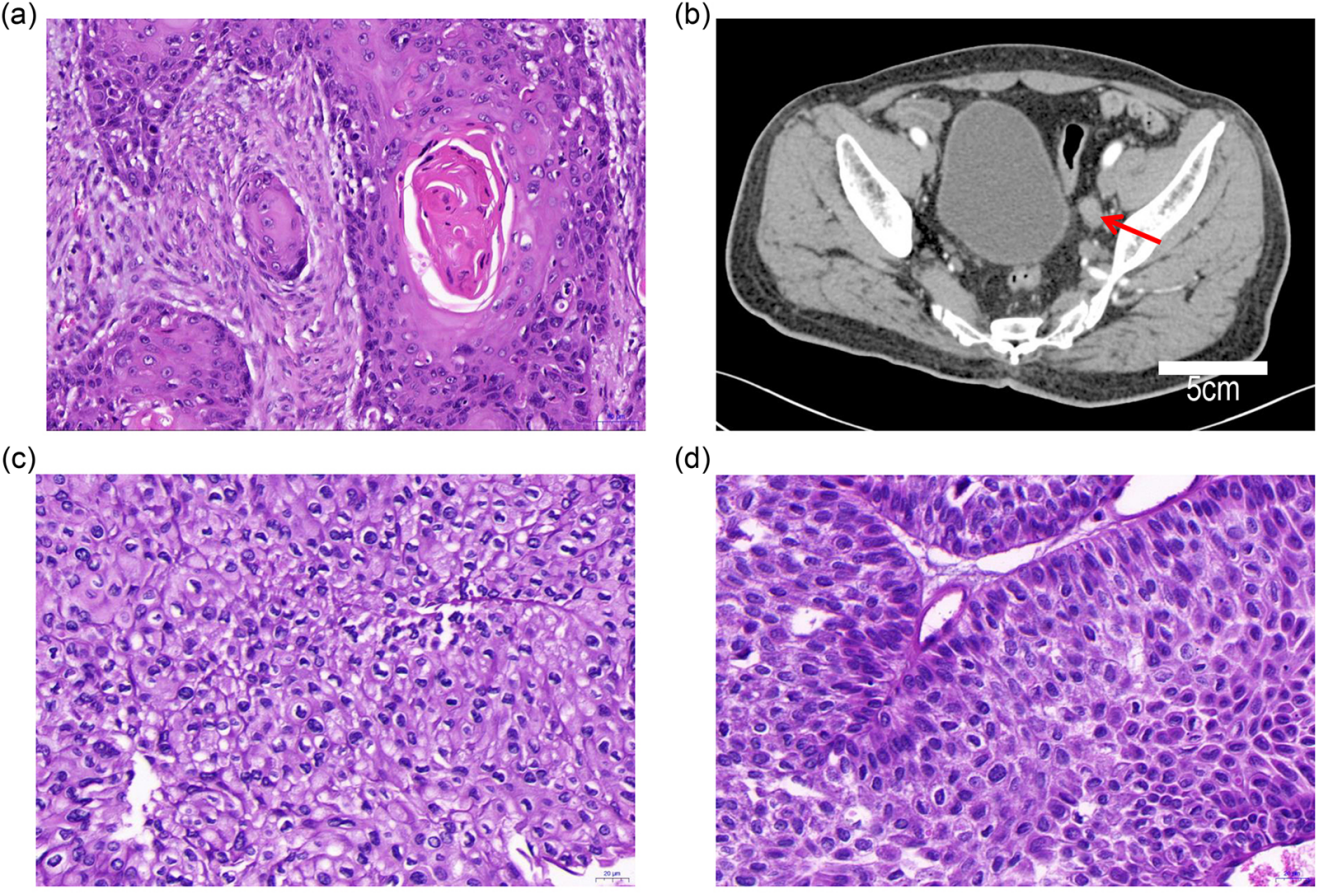
Observation of esophageal carcinoma and ureteral urothelial carcinoma: (a) pathologic findings of esophageal carcinoma postoperatively, (b) enhanced abdominal CT showed a left ureteral neoplasm (red arrow), contrast medium was iohexol, (c) pathologic findings of a left ureteral urothelial carcinoma, and (d) pathologic findings of bladder metastatic urothelial carcinoma.

In 1998, a 42-year-old male was admitted to the Thoracic Surgery Department of the General Hospital of Jinan Military Region due to food obstruction. A barium meal fluoroscopy showed that the lumen of the middle part of his esophagus was narrow with a mucosal injury; the length of the lesion was approximately 7.5 cm. A transthoracic radical resection of the esophageal tumor and an esophagogastric thoracic anastomosis were performed. Postoperative pathologic evaluation revealed a grade II ESCC with infiltration into the adventitia. The resection margins were negative in the upper and lower tangent lines, and 0 of 6 peripheral lymph nodes had tumor metastases (Figure 2a). The patient was treated with six cycles of cisplatin plus fluorouracil (the dose was unknown), and had regular follow-up thereafter.

In July 2011, he visited the hospital for painless hematuria. An enhanced computed tomography (CT) of urinary system showed that the left renal pelvis was spherical and dilated, with a long diameter of approximately 7.8 cm. A soft tissue density was noted in the lower end of the walking area (Figure 2b). He then underwent a left lower ureterectomy and ureterocystostomy. Moderately differentiated metastatic epithelial carcinoma invading the muscularis propria was confirmed by pathologic evaluation. No cancer was detected at either end of the tangent line (Figure 2c). Intravesical gemcitabine (1,000 mg qw × 8 cycles) was infused for 1 year. Follow-up evaluations were performed monthly. In March 2013, a bladder mass was detected by Doppler ultrasound, but not enhanced CT of the urinary system. A cystoscopic bladder mass resection was performed. The pathologic diagnosis was urothelial carcinoma (Figure 2d). Bladder metastases from ureteral cancer were diagnosed based on the medical history. Because the patient had received gemcitabine infusion chemotherapy, pirarubicin (20 mg/m^2^ qw × 8 cycles) intravesical instillation chemotherapy was administered for 1 year. Additional instillations were administered (20 mg/m^2^ qm × 10 cycles). Periodic re-examinations were performed after the infusion chemotherapy was completed.

**Figure 3:**
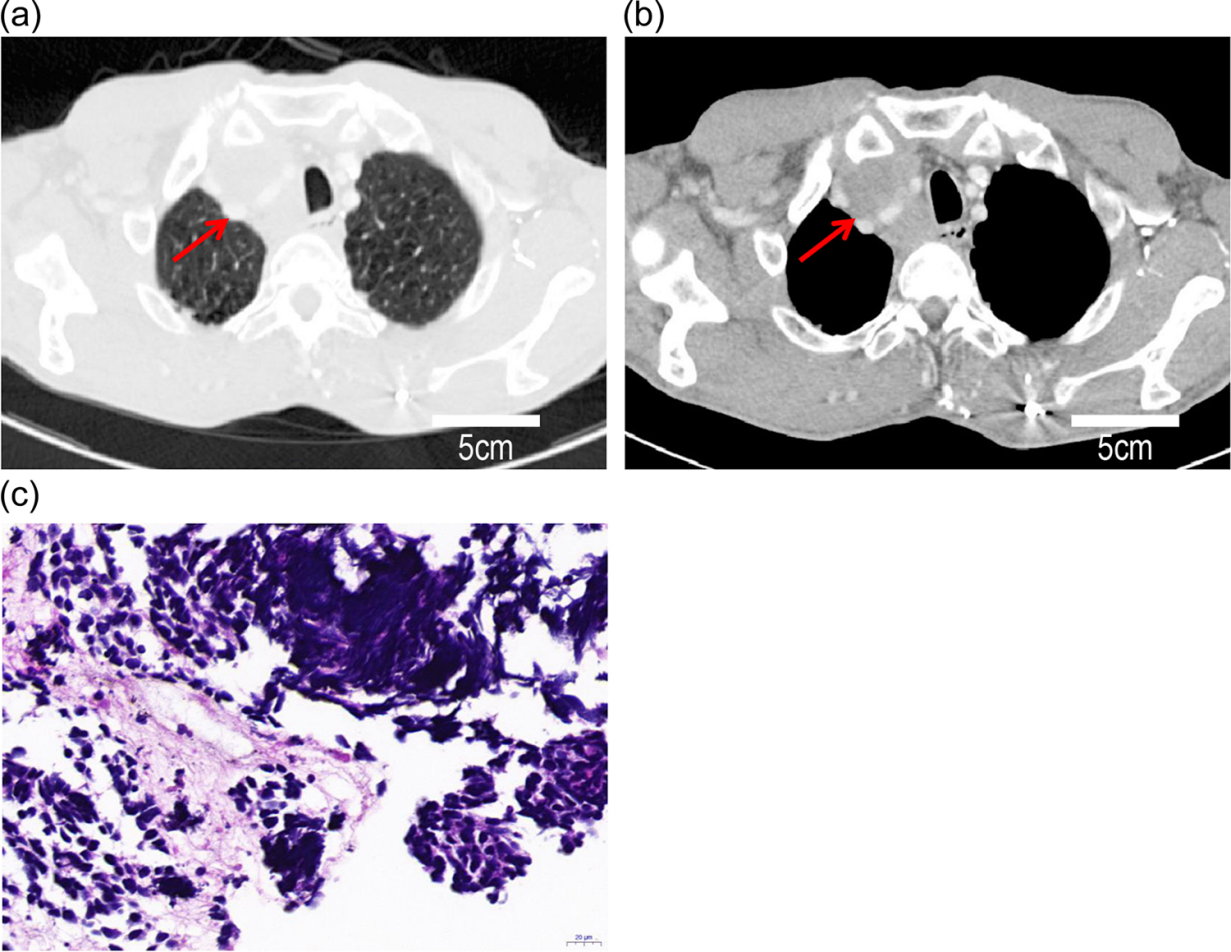
Observation of small cell cancer: (a and b) enhanced chest CT scan findings of a right upper mediastinal mass, contrast medium was iohexol and (c) pathologic findings of a right upper mediastinal mass (small cell cancer).

In June 2019, a chest CT revealed multiple mediastinal lymphadenopathy (Figure 3a and b). Given the history of esophageal cancer, metastases from esophageal cancer and a recurrence were suspected. Gastroscopy showed that the esophagus was compressed and the mucous membranes were erythematous and inflamed. An endoscopic ultrasonography guided fine needle aspiration was performed and small cell carcinoma was confirmed based on pathologic examination (Figure 3c). Combined with the imaging data, mediastinal SCLC was considered (limited stage, cT0N3M0, stage IIIB [AJCC, 8th edition]). He received etoposide (100 mg/m^2^ d1-3) and cisplatin (40 mg/m^2^ d1-2q21d) for six cycles with concurrent intensity modulated radiotherapy (60 Gy/30F). The therapeutic evaluation was a partial response according to the RECIST 1.1 standard treatment effect evaluation after treatment. Regular follow-up evaluations were performed after treatment.

**Figure 4:**
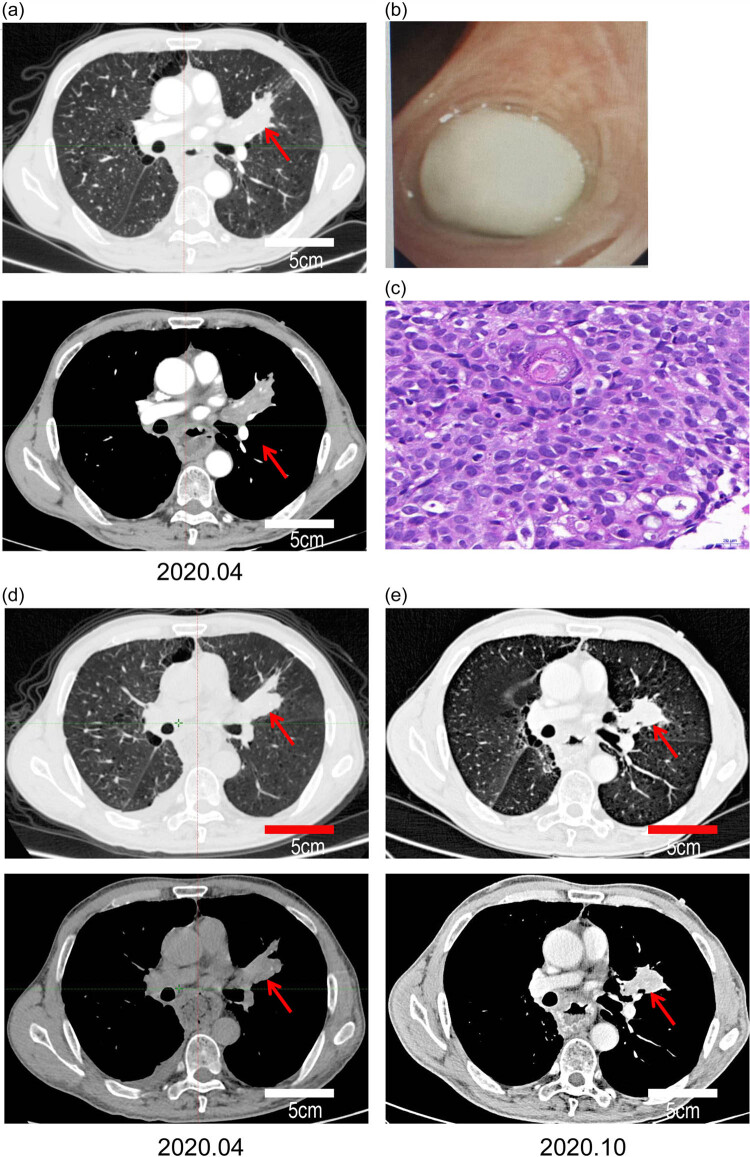
Observation of LUSC: (a) enhanced chest CT scan findings of left lung mass, contrast medium was iohexol, (b) findings of fiberoptic bronchoscopy (mass in the lingual segment of the left upper pulmonary lobe), (c) pathologic findings of the LUSC, and (d and e) chest CT scan of the LUSC after treatment with apatinib, (e) enhanced chest CT, contrast medium was iohexol.


In February 2020, he sought evaluation for blood in the sputum in our department. A enhanced chest CT showed that there was an irregular tissue mass in the lingual section of the left upper lobe near the hilum (Figure 4a). Bronchofibroscopy showed a nodular mass in the left lingual lobe bronchial opening obstructing the lumen. The surface of the mass was covered with milky white necrotic material (Figure 4b). The pathologic evaluation of the biopsy specimen revealed squamous cell carcinoma (Figure 4c). He was not considered to be a surgical candidate because the mass was too close to the hilum. The patient declined simultaneous radiotherapy and chemotherapy. Apatinib mesylate is an anti-angiogenesis medication that has a curative effect on many types of tumors. The patient began to take apatinib mesylate orally in February 2020. By October 2020, he was shown to have stable disease based on regular follow-up evaluations (Figure 4d and e).

**Informed consent:** Informed consent has been obtained from all individuals included in this study.
**Ethical approval:** The research related to human use has been complied with all the relevant national regulations, institutional policies and in accordance with the tenets of the Helsinki Declaration, and has been approved by the authors’ institutional review board or equivalent committee.

## 3 Discussion

MPMNs defined as the occurrence of two or more primary malignant tumors have increased malignant behavior and a worse prognosis in comparison with a single primary tumor [6–8]. Si et al. [9] investigated the clinical and pathological features of MPMNs cases and found that primary neoplasms consisting of gastric cancer, head and neck cancer, esophageal cancer, and colon cancer occupied most cases in male MPMNs, while primary breast cancer ranked first in female MPMNs. Copur and Manapuram [10] reported that multiple primary tumors (at least two tumors) accounted for 6.3% of all cancer patients. Further analysis showed that 2–12% of patients with two tumors continued to develop a third or fourth tumor. This case report described four primary tumors (ESCC, UTUC, SCLC, and LUSC) with separate pathologic features and explored sustainable and reasonable management of multiple primary tumors.

It has been reported that most patients with four primary tumors have breast and upper gastrointestinal tumors [11–13]. Squamous cell carcinoma is the most common histologic type of esophageal cancer worldwide, and has the highest incidence in Eastern Asia [14], Tai’an is a high-risk region of esophageal cancer. MPMNs frequently develop in patients with ESCC, especially in the upper aerodigestive tract [15]. It was found that approximately one in five ESCC patients who survive longer than 6 months will develop an secondary primary cancer within 15 years in a Western population [16]. Significantly higher risks for mouth/pharynx, larynx, pancreas, and leukemia as second cancers are clarified in patients with ESCC [17,18]. Multiple primary cancers simultaneously or metachronously occur with high rates, particularly in the head and neck region and in the esophagus [19,20]. In addition, alcohol consumption and smoking habits are common risk factors for several types of cancers in this region [4]. There was only one report about MPMNs with ESCC and renal urothelial cancer [21]. In this report, the patient developed UTUC after 10 years of esophageal cancer, after that SCLC and LUAC were diagnosed and treated.

As a result of advances in cancer research and treatment, the overall survival of patients after diagnosis continues to lengthen. Due to the growth in the number of cancer survivors, the long-term side effects of chemotherapy and/or radiotherapy, the increase in diagnostic sensitivity, and the continued influence of genetic and behavioral risk factors, the risk of MPMNs is also increasing. The specific mechanism underlying MPMNs has not been established. The root causes of primary cancers may include genetic, environmental, and treatment-related factors [22]. The sensitivity of individuals to carcinogenic factors is different, which is one of the important factors for the patient. At the same time, poor lifestyle, long-term smoking, alcoholism, an unhealthy diet (excessive fat intake and too little fiber intake) long-term chronic diseases that affect immune function, and prolonged life expectancy increase the probability of ≥ two primary tumors [23]. Long-term exposure to a carcinogenic environment (air pollution, nuclear radiation, ultraviolet rays, and industrial pollution) provides an important basis for the occurrence and development of tumors [24]. The occurrence of tumors has an evident familial aggregation, which is considered as the combined influence of environmental and genetic factors. The first mutation occurs in germ cells and the second mutation occurs in somatic cells after birth. Thus, genetic factors play an important role in tumorigenesis [25]. Although the main risk factors may be smoking and genes, and many theories have been proposed to explain MPMNs, the specific pathogenesis remains to be elucidated. A larger scale and additional data are needed to determine the predisposing factors and the degree of association. The most recent research has revealed new markers by which to screen high-risk patients and intervene in a timely fashion to improve prognosis [26,27]. In the future, effective chemoprevention, stricter monitoring, and essential cytogenetics and molecular research should be developed to improve prevention strategies and diagnosis and treatment of MPMNs, thereby improving prognosis. In addition, multidisciplinary treatment and individualized precision treatment strategies may help improve the prognosis of MPMNs.
